# Evaluation of the Sealing Ability of Direct versus Direct-Indirect Veneer Techniques: An In Vitro Study

**DOI:** 10.1155/2021/1118728

**Published:** 2021-12-30

**Authors:** Mewan Salahalddin Abdulrahman

**Affiliations:** University of Sulaimani, College of Dentistry, Kurdistan Region, Iraq

## Abstract

**Backgrounds:**

Marginal discoloration, microleakage, wear, and marginal fractures are all prevalent problems with composite veneers, and this scenario leads the esthetic outcome to deteriorate with time, resulting in patient discontent. *Aim of the Study*. The study's goal was to determine the marginal sealing ability of composite laminate veneers when employing two types of veneer techniques: direct and direct-indirect veneers, as well as two types of composite resin: nanohybrid and microfilled composite resin restorations, using dye penetration method.

**Materials and Methods:**

In this study, forty extracted human teeth were utilized. Following a standardized veneer preparation on the labial surface of the teeth, they were separated into two groups of 20 teeth each, using the following composite application techniques: group A: direct veneers and group B: direct-indirect veneers. Following that, each major group was separated into two subgroups of ten teeth each, based on the type of composite employed: subgroup 1 used nanohybrid composite resin, while subgroup 2 used microfilled composite resin. All of the samples were kept in distilled water, thermocycled, and soaked in 2% basic fuchsine dye. These specimens were sectioned and examined under a stereomicroscope for dye penetration at the gingival margin. The data was analyzed using independent *T*-tests using SPSS 22.

**Result:**

Using direct-indirect veneer technique with nanohybrid composite resin material resulted in the most negligible dye penetration at the gingival margin, while using direct veneer technique with microfilled composite resin material resulted in the maximum dye penetration. For both composite materials, gingival microleakage was lower when using the direct-indirect veneer technique than when using the direct technique, and the difference was statistically significant (*P* < 0.05). In both techniques, gingival microleakage was lower with nanohybrid composite than with microfilled composite, and the difference was statistically highly significant (*P* = 0.001).

**Conclusion:**

The sealing ability of the gingival margin of tooth/composite interface is better when applying direct-indirect veneer technique with nanohybrid composite resin than that of direct veneer technique with microfilled composite resin material.

## 1. Introduction

In today's dentistry, a patient's esthetic look is essential [1]. The adoption of conservative restoration solutions to restore the esthetic look of the dentition has increased as adhesive techniques have improved [2]. Following recent advancements in adhesive and restorative dentistry, direct resin veneers have become one of the most prevalent treatment alternatives for clinical applications [3]. These restorations are directly put with an adhesive agent and a composite resin material on minimally prepared or even unprepared tooth surfaces in a single dental clinic visit [4]. Direct laminate veneers have the advantage of allowing the operator to control and evaluate the restorative process from shade selection to final morphology [5], as well as better marginal adaptation, easy intraoral polishing, low cost, no need for an additional adhesive cementing system, and easy repairability [6].

The endurance and durability of direct composite resin restorations are multifactorial, and the triad of material, technique, and the operator must be considered when evaluating failure causes [7]. On the other hand, these materials can produce outstanding and long-lasting results when correctly selected and handled [8]. In clinical investigations, minor discoloration has been linked to patient unhappiness with veneers. It has also been thought to be proof of a minor flaw, such as partial debonding or microleakage [9]. The importance of marginal leakage in the maintenance of dental esthetics cannot be overstated [2]. Because of the differences in physical qualities between teeth and restorative materials, gaps arise at the tooth/restoration interface, resulting in marginal microleakage, which is the leading cause of failure in dental esthetic composite restorations [10].

The direct-indirect veneer technique, also known as (semidirect) veneer technique, is a modified veneer application technology that combines the benefits of both direct and indirect veneer techniques [5, 11]. When this technique was first introduced, the main advantages emphasized the ability to expose intraorally made veneers and inlays to extraoral light and heat to optimize their physical and mechanical properties, clinical behavior, excellent esthetics, and unrivaled marginal adaptation and polishing [12].

Microfills were the most common composites used for veneering at first, owing to a desire to emulate the reflectivity of the enamel surface [13]. Microfill has been proved to be the only composite material that can withstand the test of time in terms of color stability and polishability [14]. On the other hand, nanohybrid composite is a popular direct veneer material because it incorporates nano- and microsized filler particles that provide good mechanical strength as well as finishing and polishing results and a relatively smooth surface and a high gloss that are similar to porcelain restorations [15] As a result, we used two types of composite resin in this study: microfilled and nanohybrid composite restorations and two veneer application techniques: direct veneer and direct-indirect veneer to assess the gingival marginal sealing ability of composite laminate veneers using the dye penetration method. To our knowledge, no research has been done to compare the marginal sealing performance and microleakage of direct veneer versus direct-indirect veneer techniques.

## 2. Materials and Methods

The present study was approved by the Ethics Committee of Dentistry College at University of Sulaimani (No. 30/21 on 24/5/2021).

### 2.1. Sample Calculation and Sampling Power

Based on the below formula, the sample size was 40 and the power of the study was 90%.

The sample size is based on the following equation:

(1) $$\begin{array}{c} n\frac{\left(Z\alpha +Z\beta \right)2*2*S2}{\left(d\right)2},\\ n=\frac{\left(1.96+1.28\right)2*2*0.2682}{\left(0.536\right)-0.332}. \end{array}$$

*n* = 37, so we chose 40. *n* is the sample size, which is 2 as we have two methods of comparison. *S* is the standard deviation of the variable under the study (taken from a previous study which was 0.268). *d* is the difference the investigator wishes to detect taken from the difference of two means of the same study (0.536 − 0.333) = 0.203. ά is the conventional multiplier for alpha = 0.05. *Z*ά = 1.96. 1 − *β* for power of the study 90%, so *Zβ* is 1.28.

### 2.2. Selection of Teeth

**Figure 1:**
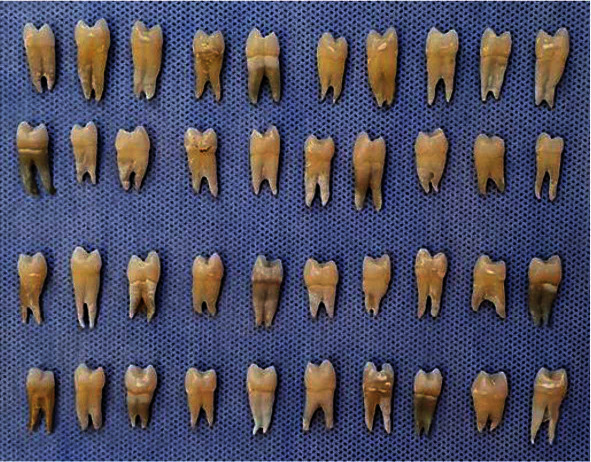
The collection of teeth.

This study included forty extracted intact human upper first premolars with no caries, fissures, or severe wear (extracted for orthodontic reasons) (Figure 1). Before the trial, all teeth were cleaned of debris and calculus and preserved in normal saline to ensure that dentinal permeability and composite veneer binding strength were unaffected. The mesiodistal and incisocervical labial surfaces were measured using a digital caliper with an accuracy of 0.01 mm to check that the measurements of the teeth were similar. The teeth that were chosen had dimensions that were within 1 mm of the average.

### 2.3. Division of Groups

**Figure 2:**
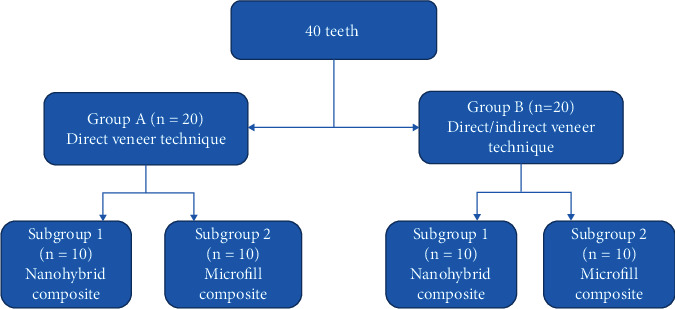
The division of groups.

According to the composite application techniques, the teeth were randomly divided into two major groups: group A: direct veneer technique (*n* = 20 teeth) and group B: direct-indirect veneer technique (*n* = 20 teeth). The composite utilized was then used to separate each leading group into two subgroups (*n* = 10 teeth). Nanohybrid composite resin (Tetric N-Ceram, Ivoclar Vivadent, Liechtenstein) was utilized in subgroup 1 (*n* = 10), while microfilled composite resin (Renamel Microfill, Cosmedent, Chicago, IL) was used in subgroup 2 (*n* = 10). The division of the groups is depicted in Figure 2. For standardization, the A1 composite shade was chosen.

### 2.4. Surface Preparation of Teeth

**Figure 3:**
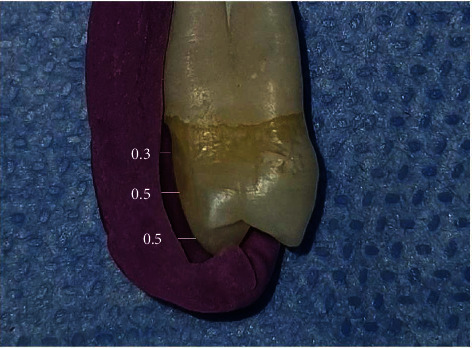
Veneer preparation outline; a silicone index was used to evaluate tooth reduction accuracy.

Before the preparation, putty condensation silicone imprint material (Zetaplus, Zhermack, Italy) was used to construct a silicone index for each tooth in all groups to check the accuracy of tooth reduction shown in Figure 3. After that, a particular laminate veneer preparation bur set was used to prepare the teeth (Laminate Veneer Set, Axis, Kerr, Texas, USA). To guarantee that the veneer was adequately sealed, a chamfer finish line had to be within the enamel. As shown in the illustration, diamond depth cut burs (M834016, M834021, Axis, Texas, USA) were used to scribe horizontal depth cut grooves on the labial surface for minimal preparations of approximately 0.3 mm in the cervical third and 0.5 mm in the middle and incisal third (Figure 3). The chamfer finishing line was inserted in the cervical margin below the cementoenamel junction about 1 mm toward the occlusal surface, limited in enamel along the incisal edge without shortening it, and positioned in the interproximal embrasures without breaking contact. After that, a diamond rotary cutting equipment (H284K016) was used to link all of the grooves. The surface was prepared with a retouch bur in the middle third (SF134014), in the cervical third (SF132008), and in the incisal third (SF379023).

### 2.5. Veneer Application Technique

#### 2.5.1. Group A: Direct Veneer Technique

*(1) Application of Composite Resin*. After dental surface preparation, 37% phosphoric acid (Prime Dent, Etchant Gel, Chicago, IL, USA) was applied to the enamel surfaces for 15 seconds, washed for 20 seconds, and slightly dried. The bonding agent (3M ESPE, Adper, Single Bond Plus Adhesive, USA) was applied in two layers using a bonding brush (TPC, Dental Disposable Micro Prophy Brush, HTY, Henan, China) on the prepared tooth surfaces, with the excess removed with oil-free air spray and polymerized for 20 seconds with a light-curing unit (Optilux 500, Demetron/Kerr, Danbury, CT).

The selected composites for each subgroup were applied to the labial tooth surface using a stainless steel nonadherent spoon-end-shaped composite instrument (Almore, Portland, OR), brush (#400, Takanishi, Renfert, Hilzingen, Germany: #1, #2 fine-tipped, and #3 flat-tipped, Cosmedent, Chicago, IL), and contouring instrument using an Optrasculpt pad, with gentle digital pressure. We utilized a layering technique in this procedure, without using any bonding agent between the layers, and polymerized for 20 seconds using a light-curing unit (Elipar FreeLight II, 3M ESPE, USA) placed 3 mm away from the resin composite surface.

*(2) Contouring, Finishing, and Polishing*. Finishing was achieved with aluminum oxide disc (Sof-Lex Pop-On XT, 3M, St. Paul, MN), a combination of medium-grid diamonds (#859-081 and #856-L-016, Brasseler, Savannah, GA), and #12-fluted carbide finishing bur (#7901, S.S. White, Lakewood, NJ) was used to remove gross composite excess resin around the incisal and the embrasures. The surface was texturized with a medium-grid taper diamond bur (6856L-016, Brasseler, Savannah, GA).

For initial polishing, rubber polisher rotaries with various degrees of abrasiveness (FlexiCups, Cosmedent, Chicago, IL) were employed sequentially. Then, use a buffing disc and a polished composite paste (Foto-Gloss, Kota, Sao Paulo, Brazil) (Flexibuff, Cosmedent, Chicago, IL). Finally, to finish and polish the margins to an optimum shape, smoothness, and shine, we utilized polishing disks with varying grain sizes (medium, fine, and extrafine) (Sof-Lex, 3M/ESPE, St. Paul, MN, USA) for 20 seconds each, using a low-speed handpiece with circular motions and without water cooling [5].

#### 2.5.2. Group B: Direct-Indirect Veneer Technique

*(1) Application of Composite Resins*. The composites selected for each subgroup of this group were initially applied to the labial tooth surface (without acid etching or bonding) with a stainless steel Heidemann spatula, brush, and contouring instrument equipped with an Optrasculpt pad, using gentle digital pressure to create the primary contour of the restoration. In this method, we utilized a layering strategy without using a bonding agent, followed by a 20-second light cure, Fahl [5] and Fahl and Ritter [11] outlined the step-by-step procedure for direct-indirect veneer.

*(2) Veneer Removal*. Using the thin-bladed end of a composite instrument (Goldstein Flexi-Thin Composite Instruments, Mini #3, HuFriedy, Chicago, IL), the veneer was gently removed to avoid fracture. Next, it was inserted at the veneer/tooth contact at the faciogingival embrasure level using an excavator (Bader, 17-4024-15), and moderate, but firm pressure was applied to both the mesial and distal sides using a leveraging motion.

**Figure 4:**
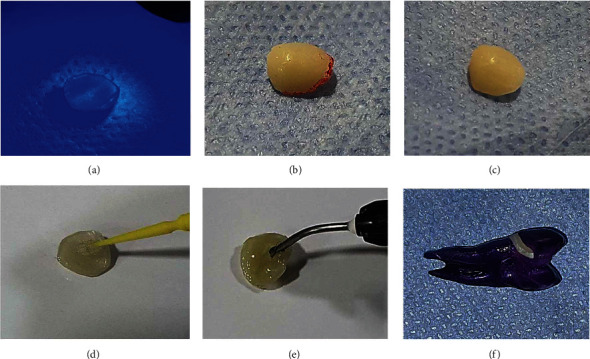
(a) The finished veneer is submitted to supplemental extraoral light curing. (b) The imprinted margins are outlined with a red pencil. (c) The margins after finishing and polishing to ideal contour. (d) The inner surface of the veneer is silanated with silane liquid material. (e) The inner surface of the composite veneer is loaded with light-cured luting cement. (f) The specimens are coated with two layers of nail varnish except for a 2.0 mm rim around the laminate veneer gingival margins.

*(3) Supplementary Extraoral Light Curing and Heat Tempering*. The completed veneer was then exposed to supplemental extraoral light curing for 20 seconds (Figure 4(a)) and heat tempered for 15 minutes at 121°C using an autoclave (Zirbus Technology GmbH, Bad Grund (Harz), Germany).

*(4) Contouring, Finishing, and Polishing*. After the composite veneer had been heated, it was removed, and the imprinted borders were drawn with a red pencil, as illustrated in Figure 4(b). Aluminum oxide discs (blue color, Tor Vm, polishing discs, Moscow, Russia) were used consecutively to remove excess material and finish and polish the margins to an optimum shape (Figure 4(c)), smoothness, and shine. Following that, the veneer was repositioned on the tooth and tested for fit and stability. The incisal one-third was then flattened until the angle between the facial-incisal line and the face volume and incisal length was correctly aligned. Following that, the veneer was seated, and then, emergence profile and face planes were created (before bonding).

Then, using course discs to anatomically blend the cervical and incisal thirds, create the proper face shapes. Next, the veneer was removed, and the facial embrasures were completed to their correct morphology. Finally, finishing and polishing were performed using discs (Sof-Lex, 3M/ESPE, St. Paul, MN, USA) with various grits (medium, fine, and extrafine) for 20 seconds each, using a low-speed handpiece with circular motions and without water cooling, until the primary anatomy of the veneer was attained.

*(5) Tooth Surface Treatment*. The tooth surface was etched with 37 percent phosphoric acid applied to the enamel surfaces for 15 seconds, washed with water spray for 20 seconds, and gently dried. Following that, a bonding agent was applied in two layers to the prepared tooth surfaces using a bonding brush; the excess was cleaned with an oil-free air spray, and the bonding agent was polymerized for 20 seconds with a light-curing unit.

*(6) Veneer Cementation*. The inner surface of the veneer was cleaned with alcohol and dried and then acidified with 37 percent phosphoric acid for 10 seconds and silanated with silane liquid material (Monobond Plus, Ivoclar Vivadent, Liechtenstein, Germany) as shown in Figure 4(d), followed by the application of a bonding agent according to the manufacturer's recommendation. It was, moreover, thinned with air. The adhesive bonding agent was not light-cured before seating the completed restoration in this method since it may pool if not thinned properly, resulting in a thicker layer that inhibits the veneer from fitting precisely. Finally, the veneer was left aside, being covered by a light protective cover till it was luted.

After applying the composite veneer with light-cured luting systems (Variolink, Veneer, Ivoclar Vivadent Inc., Liechtenstein, Germany), it was seated on the tooth surface and held in place with finger pressure, and the marginal excesses of the luting material were removed with a blunt, rubber-tip instrument (GUM Stimulator, Sunstar) to allow some cement to remain at tooth restoration. With an explorer, the margins were examined for the precision of fit to ensure the veneer was utterly placed in the correct location. Following that, the veneer was light-cured from the labial surface for 120 seconds using a light-curing device [5]. Additional access cement was removed using the laminate system veneer kit's #12 blade and ultrafine diamond bur. Composite veneers were polished and treated in the same manner as to direct veneers in group A.

#### 2.5.3. Evaluation of Marginal Microleakage

The specimens were then thermocycled 500 times between 5 ± 2°C and 55 ± 2°C with a dwell period of 30 seconds in each bath and a 20 s gap between baths at ambient air temperature. Following that, the specimens were covered with two layers of nail varnish (Orly International Inc., California, USA), except for a 2.0 mm rim around the laminate veneer gingival margins to allow dye contact with the veneer margin, as illustrated in Figure 4(f).

**Figure 5:**
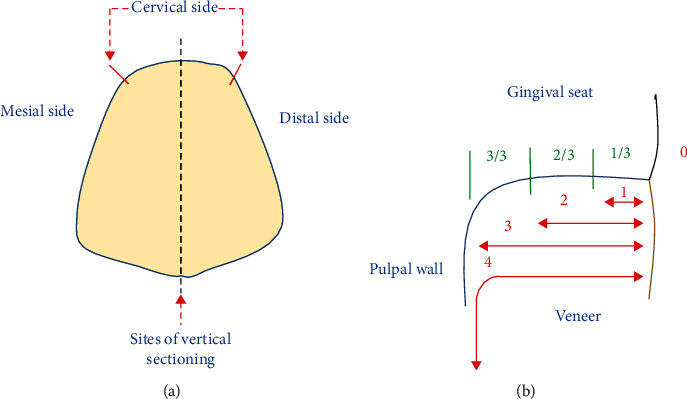
(a) The side of vertical sectioning. (b) The dye microleakage scores.

The specimens were then immersed in a 2 percent basic fuchsine dye solution for 24 hours and then withdrawn from the dye, rinsed with tap water, and dried for a further 24 hours according to the manufacturer's instructions. Later, the specimens were sectioned buccolingually into two halves parallel to the tooth's long axis using a diamond disc (15LC Diamond Wafering Blade, Buehler) and a water-cooled diamond saw at a modest speed. The section was applied in the middle of the mesiodistal dimension of the cervical margin of the veneer (from the mesial interproximal embrasures to the distal interproximal embrasures point), as shown in Figure 5(a).

All samples were evaluated blindly by two independent evaluators using a stereomicroscope (Olympus SZ 60, Japan) at a magnification of 40 on a five-point scale to determine the degree of dye penetration along with the laminate-tooth interface at the gingival margin. The microleakage scores were as follows [16] (Figure 5(b)):

0 = no dye penetration

1 = dye penetration up to the first third of the gingival seat

2 = dye penetration up to the second third of the gingival seat

3 = dye penetration into the entire gingival seat

4 = dye penetration into the entire gingival seat and the pulpal wall

#### 2.5.4. Evaluation of the Microleakage Percentage

Additionally, another way was used to assess the microleakage in the specimens, by calculating the percentage of the microleakage depending on the dye penetration distance. The specimens were evaluated using a digital microscope (VHX 600, Keyence, Osaka, Japan), at 30x magnification power. An image analysis software was used to assess the leak by measuring the extent of the penetration of the dye which was calculated by measuring the distance from the external surface to the spot where no dye can be detected in *μ*m. To comply with the areas that are included in the scores, a total of 400 *μ*m of the distance was examined from the external edge to the pulp floor, and each 100 *μ*m was correlated with the corresponding score in which that distance is included. This was done to facilitate the comparisons of the results obtained from both techniques. The percentage of the microleakage was calculated by the following formula:

(2) $$\begin{array}{c} \text{Microleakage}\%=\frac{\text{Linear distance of penetration of the dye}}{\text{Total linear distance from the external margin to the pulp floor}}\times 100. \end{array}$$

## 3. Statistical Analysis

The collected data was recorded by the dye penetration index (0–4) and then subjected to the Microsoft Excel and transferred to SPSS program version 22 (SPSS ver. 22, SPSS IBM SPSS; Chicago, IL, USA). We used compared mean independent *T*-test and found mean and standard deviations and then *P* value, in which *P* ≤ 0.05 was regarded as statistically significant.

## 4. Result

The score, percentage, and range for the microleakage in each of the groups and subgroups are shown in Table 1. The mean and standard deviation (SD) are shown in Table 2, indicating that the most negligible dye penetration was observed in group B when using the direct-indirect veneer technique with a nanohybrid composite resin material (0.1 ± 0.32) (0.22%). In contrast, the most dye penetration was observed in group A when using the direct veneer technique with microfilled composite resin material (1.7 ± 1.33) (31.63%).

The mean dye penetrations of nanohybrid and microfilled composites were compared when both veneering techniques were used; in group A, when the direct veneering technique was used, the microleakage of the nanohybrid composite was significantly less than that of microfilled composite, with the difference being statistically highly significant (*P* = 0.001). When the direct-indirect veneering technique was used in group B, the microleakage of the nanohybrid composite was also less than that of the microfilled composite, and the difference was statistically highly significant (*P* = 0.001), as demonstrated in Table 3.

The mean dye penetrations of direct veneer and direct-indirect veneer were also compared when both types of the composite were used; in subgroup 1, when nanohybrid composite was used, the microleakage in the direct-indirect veneer was less than that in the direct technique, and the difference was statistically significant (*P* < 0.05). Additionally, when a microfilled composite was used in subgroup 2, the microleakage in the direct-indirect veneer was smaller than that in the direct approach, and the difference was statistically significant (*P* < 0.05) (Table 4).

**Figure 6:**
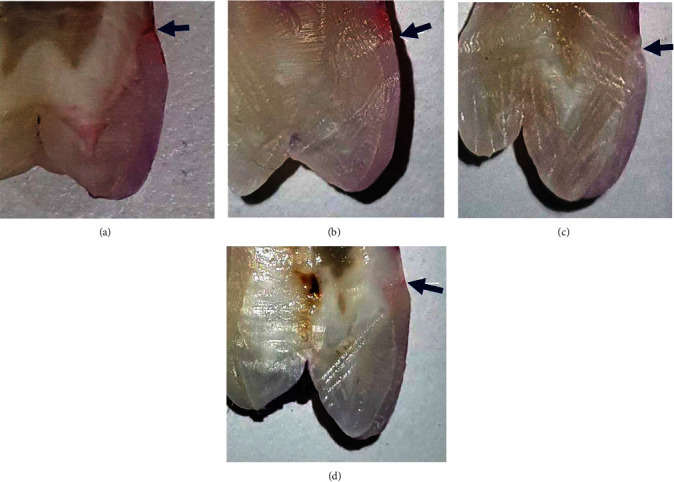
Tooth samples demonstrating dye penetration along the laminate-tooth interface at the gingival margin. (a) One sample from group A, subgroup 1, when using nanohybrid composite with direct veneer technique. (b) One sample from group A, subgroup 2, when using microfilled composite with direct veneer technique. (c) One sample from group B, subgroup 1, when using nanohybrid composite with direct-indirect veneer technique. (d) One sample from group B, subgroup 2, when using microfill composite with direct veneer technique.

The dye penetration along the laminate-tooth contact at the gingival margin is depicted in the different groups (Figure 6).

## 5. Discussion

Composite laminate veneers are one of the least intrusive treatment options available for rejuvenating and restoring a patient's smile; they can be glued to the tooth structure and replicate the optical features of natural teeth esthetically [17]. However, marginal discoloration, microleakage, wear, and marginal fractures are all prevalent problems with composite restorations, and this circumstance results in a gradual deterioration of the esthetic result [2]. Microleakage is characterized as the dynamic, clinically undetected flow of bacteria, fluids, chemicals, compounds, and ions between the cavity walls and the applied restorative material [18]. Microleakage can be caused by a variety of circumstances, including the resin material adapting to the tooth surface “often at the gingival margin,” the adhesive technique utilized, and material polymerization shrinkage [19, 20]. However, appropriate adhesion between the restorative material and the cavity walls results in effective marginal sealing, reduced microleakage, decreased discoloration, and a more prolonged life restoration [21]. Thus, the direct-indirect technique evolved, combining many of the advantages of the direct and indirect techniques and promising to improve the physical and mechanical properties of the extraoral chairside tempering process due to increased monomer conversion and decreased polymerization shrinkage [22].

Numerous factors influence the esthetic quality and endurance of dental restoration, including the technique used to install the restoration, the degree of polymerization of the composite, and the type of restorative material employed [20]. Thus, the purpose of this study was to determine the effect of the composite laminated veneer application technique (direct and direct-indirect veneer) as well as the effect of the composite resin type (nanohybrid and microfilled composite) on the gingival marginal microleakage of the composite laminated veneer interface.

Currently, a variety of different types of composite are available on the market, some of which are intended for use in areas with a greater emphasis on esthetics (anterior teeth). In contrast, others are intended for use in areas where more excellent resistance to masticatory forces is required (posterior teeth) [23]. The amount and size of fillers have been shown to affect the surface roughness and stain resistance, with microfilled composites demonstrating superior superficial behavior, superior surface sheen, decreased marginal and surface staining, improved color match, and superior marginal adaption [24]. Additionally, nanohybrid composites promise to improve physical and optical properties, resulting in easier and longer-lasting polishability, improved shade matching and light reflection/deflection properties for natural esthetics, and decreased shrinkage for high marginal integrity and decreased likelihood of microleakage and postoperative sensitivity [25]. As a result, we chose those two varieties of esthetic composite resin materials for this study.

Microleakage testing is used to predict the performance of restorative materials in the oral environment by clinicians and researchers [18]. Dye penetration is a regularly used technique for determining marginal microleakage between the material and the tooth structure as well as the sealing ability [19]. Numerous researches on edge leakage have employed the primary fuchsine dye penetration method to determine edge leakage. This straightforward method determines whether or not there is a leak [16, 26, 27].

Regardless of the composite type, the results of this study indicated that group B experienced less microleakage when using the direct-indirect veneer approach than group A when using the direct veneer technique. This is because the final strength of the tooth-restoration complex is significantly dependent on adhesive techniques when using the direct-indirect approach [28]. Additionally, this approach minimizes polymerization shrinkage since the polymerization happens outside the cavity, leaving only the resin cement to contract and connect the restoration to the tooth structure [29]. To clarify further, with the direct-indirect procedure, the restoration is sculpted directly into the tooth structure and removed following light activation; it can then be thermally treated, completed, and polished before adhesion and luting processing. The purpose of the extraoral light curing and heat tempering is to enhance monomer conversion while avoiding detrimental pulp overheating in essential teeth. As a result, the final restorations have superior mechanical qualities and unmatched marginal adaptation and polishing.

Additionally, the gap that may occur due to the restorative resin's polymerization shrinkage in a straightforward procedure is accounted for by a precise adaption of the directly sculpted veneer in conjunction with a thinner film of resin luting agent [11]. While in the direct composite veneer approach, polymerization shrinkage happens to a small extent. However, when polymerization happens indirectly, shrinkage is limited to the width of the luting area, which may minimize the harmful effects at the interface [30]. Due to the polymerization of monomers, the composite shrinks during polymerization. Where polymerization shrinkage is most significant, the composite adhering to the tooth produces a gap, resulting in the production of microleakage. Thus, the effectiveness of cavity restoration with resin composite material is contingent upon the restorative material's close adhesion to the cavity formed [31]. Additionally, the difference in thermal and contraction coefficients between the tooth structure and the restorative material applied had been implicated in microleakage via marginal percolation or disruption of the marginal enamel etch bond, allowing microleakage in the space created by thermal contraction [18].

On the other hand, we used two types of composite resin in this study: nanohybrid and microfilled composite resins, as they are routinely used in esthetic dental practice due to their advantages. For example, in microfilled composite, the filler is amorphous silica particles with an average diameter of 0.04 mm and a particle size of less than 1 *μ*m. The primary properties of these composites are their ability to develop and retain a high polish over time and their outstanding enamel-like translucency [32]. At the same time, nanohybrid composites use nanoparticles with a diameter of 0.005-0.01 *μ*m in conjunction with more conventional filler technologies. Nanotechnology enables more polishability, while increasing particle size results in increased strength and easier shade selection system, fluorescence, radiopacity, translucency, and improved handling [33]. As a result of their exceptional esthetic and wear properties, polishability, and handling characteristics, they are gaining popularity [34].

The dye penetration was shown to be less when nanohybrid composites were used in subgroup 1 than microfilled composites in subgroup 2, particularly when employing the direct-indirect composite veneer technique. To our knowledge, no study has been conducted to compare the two types of composite in direct-indirect or in direct veneer technique. However, a study by Shah et al. [35] discovered that microleakage in the enamel-composite interface of class I cavity preparation was less when nanohybrid composite was used versus microfilled composite. Polymerization shrinkage and rheological properties of a composite are determined mainly by the monomer types used and the ratio of resin matrix to inorganic filler (type and quantity) [36]. Moreover, because microfilled composites contain a lesser percentage of filler, their physical qualities are inferior to those of hybrid composites; the exception is their compressive strength, which can be relatively high. Microfilled composites have increased thermal expansion coefficients, increased water absorption, increased polymerization shrinkage, decreased modulus of elasticity, decreased tensile strength, and decreased fracture toughness [37]. Additionally, with smaller filler volume, there is a greater likelihood of restorative material contracting and volumetric contraction away from the tooth surface, as with microfilled composite, which results in insufficient adaptation of materials and incomplete marginal sealing. As the adaptation tool is removed from the cavity, the composite with less filler tends to be dragged away and provides minimal resistance to placement forces due to its sticky nature [38]. On the other hand, because nanohybrid composites are composed of large agglomerated nanoclusters, they provide the composite with densely packed, nanosized particles that provide a wear-resistant surface while also keeping the composite paste fluid easy to work with, thereby improving handling and esthetic properties [34]. Finally, since increased filler loading leads to less polymerization shrinkage, nanohybrid composites exhibit much lower marginal leakage than more traditional filler technologies [39]. Since the preparation was made in enamel (0.3 mm in cervical third and 0.5 mm in the middle and incisal third), then it would be suggested to use phosphoric acid and then apply only 3M™ Adper™ Scotchbond™ Multipurpose Adhesive Refill; hence, the use of three-step etch-and-rinse technique could achieve more positive results in future studies.

The main limitation of this study was that it was performed in laboratory conditions; however, the best way to test restorative materials would be in the oral cavity. In addition, the composite laminate veneers were not exposed to mechanical cycling, and all groups were exposed only to thermocycling in the laboratory. Furthermore, thermocycling itself may increase the microleakage [40]. Further studies of long-term marginal sealing are required to confirm the results of the present study and use different dental composite materials for anterior tooth veneer restorations.

## 6. Conclusion

This study found that using a direct-indirect veneer technique had a significantly less microleakage and a better sealing ability than a direct veneer technique. Meanwhile, combining direct-indirect veneer technique with nanohybrid composite resin showed superior results to using direct-indirect veneer technique with microfilled composite resin.

## Figures and Tables

**Table 1:** Microleakage score, percentage, and range for both techniques and composite resin groups.

Types of veneer technique	Types of the composite	Microleakage score and percentage						
		0	1	2	3	4	Total	Range percentage of microleakage
Group A Direct veneer technique	Nanohybrid composite	5 0%	4 7.68%	1 28%	0 0%	0 0%	10 5.88%	0–28%
	Microfilled composite	2 0%	3 15.66%	2 38.75%	2 56.25%	1 79.25%	10 31.63%	0–79.25%
Group B Direct-indirect veneer technique	Nanohybrid composite	9 0%	1 2.25%	0 0%	0 0%	0 0%	10 0.22%	0–2.25%
	Microfilled composite	8 0%	2 9.75%	0 0%	0 0%	0 0%	10 1.95%	0–13.25%

^∗^The percentage in each score indicates the mean percentage of the microleakage in that score. ^∗^The percentage in each total indicates the mean percentage of the microleakage in that group.

**Table 2:** The mean and SD of the groups.

Types of veneer technique	Types of the composite	Mean ± SD
Group A Direct veneer technique	Nanohybrid composite	0.5 ± 0.52
	Microfilled composite	1.7 ± 1.33
Group B Direct-indirect veneer technique	Nanohybrid composite	0.1 ± 0.32
	Microfilled composite	0.2 ± 0.42

**Table 3:** Comparison of gingival microleakage test between both technique groups (direct veneer technique and direct-indirect veneer technique) when using two types of composite.

Types of technique	Types of composite	Mean ± SD	*P* value
Direct veneer technique	Nanohybrid-microfilled composite	1.10 ± 1.16	0.001
Direct-indirect veneer technique	Nanohybrid-microfilled composite	0.15 ± 0.36	

**Table 4:** Comparison of gingival microleakage test between both composite resin materials (nanohybrid and microfilled composite) when using two veneering techniques.

Types of composite	Types of technique	Mean ± SD	*P* value
Nanohybrid composite	Direct/direct-indirect veneer technique	0.30 ± 0.47	0.03
Microfilled composite	Direct/direct-indirect veneer technique	0.95 ± 1.23	

## Data Availability

The data used to support the findings of this study are available from the corresponding author upon request.
